# Personalized Risk Assessment and Treatment in Ruptured Isolated Posterior Spinal Artery Aneurysm: A Rare Case and Systematic Literature Review

**DOI:** 10.3390/jpm16090462

**Published:** 2026-08-31

**Authors:** Elisa Garbin, Nicola Cavasin, Luca Basaldella, Salima Magrini

**Affiliations:** 1 Department of Neurosciences, Neurosurgery, Ospedale dell’Angelo, Mestre, 30174 Venice, Italy; luca.basaldella@aulss3.veneto.it (L.B.); salima.magrini@aulss3.veneto.it (S.M.); 2 Department of Neurosciences, Neuroradiology, Ospedale dell’Angelo, Mestre, 30174 Venice, Italy; nicola.cavasin@aulss3.veneto.it

**Keywords:** posterior spinal artery, spinal artery aneurysm, spinal subarachnoid hemorrhage, personalized medicine

## Abstract

**Background:** Ruptured isolated spinal artery aneurysms (SAAs) are not common. Posterior spinal artery aneurysms (PSAAs) are less common. Either is rarely the cause of spontaneous spinal subarachnoid hemorrhage (sSAH). Due to a few published reports, the natural course, diagnosis and optimal therapeutic strategy remain debatable and challenging. We report the case of a 60-year-old male presenting with lumbar and abdominal pain, which swiftly became associated with headache, photophobia and worsening neck pain. Spinal MRI revealed an intradural hemorrhage collection from the T9 level to the T10 level; the spinal DSA confirmed a fusiform dissecting PSAA originating from the T11 level. He was managed conservatively and recovered completely. We have reviewed the few cases reported in the literature. **Results**: Except for the present case, there are 29 reported cases of isolated PSAAs and 69 cases of isolated SAAs. The patients’ ages ranged from 16 to 88 years, and common presenting symptoms were headache and back/abdominal pain. The PSAAs were commonly located at the lower thoracic level or lumbar level. The most common PSAA treatment was surgery; among the 29 cases, 85% reported favorable outcomes regardless of the type of treatment. **Conclusions:** Ruptured spinal artery aneurysms are prone to spontaneous thrombosis. Given the lack of standardized guidelines, a personalized medicine approach is essential, requiring a detailed, patient-specific risk assessment. Conservative management could be an appropriate treatment option with a favorable outcome. Further understanding may help guide future clinical decision-making and tailored treatment planning according to patient-specific features.

## 1. Introduction

Isolated spinal artery aneurysm (SAA) is a rare cause of spontaneous spinal subarachnoid hemorrhage (sSAH) and little is known about its actual incidence or the natural history of this lesion [1,2]. Due to the rarity and the heterogeneity of this pathology, experience from various studies remains primarily limited to case reports and small-sample case-series studies [1,2].

Generally, sSAH is attributed to secondary causes such as spinal vascular malformations (AVMs) or dural arteriovenous fistula (dAVF), which are the leading causes of this clinical and radiological presentation [2]. Conversely, an isolated ruptured spinal aneurysm as the etiology of spinal SAH is much rarer [3,4]. Sometimes AVM or dAVF are associated with SAA, but isolated SAA is extremely rare and it remains a highly uncommon cause of sSAH. SAAs frequently are associated with concomitant vascular or non-vascular pathologies that impose hemodynamic stress along the spinal parent arteries, thereby inducing aneurysm formation [1,4]. Reported associations and secondary causes of sSAH include spinal AVM, dAVF, aortic coarctation, MoyaMoya disease, vertebral artery occlusion, connective tissue disorders, infections, and previous trauma [2,5,6]. Among all the causes of sSAH, less than 1% are of pure spinal origin [2,7,8,9,10,11].

The diagnosis is often challenging and delayed due to its rarity. Owing to the uncertain natural history of these lesions, there is currently a lack of consensus on the optimal treatment strategy [3,6]. We have reviewed the literature on previously reported cases of isolated SAAs and we present our experience regarding a case of an isolated PSAA at the T11 level treated conservatively. The main aim of this data analysis is to provide a detailed literature overview to better understand this rare occurrence, highlighting patient demographics, clinical features and treatment. We focused on the identification of the general features and current knowledge on the treatment of this pathology [2,5,10,11,12].

## 2. Materials and Methods

**Figure 1:**
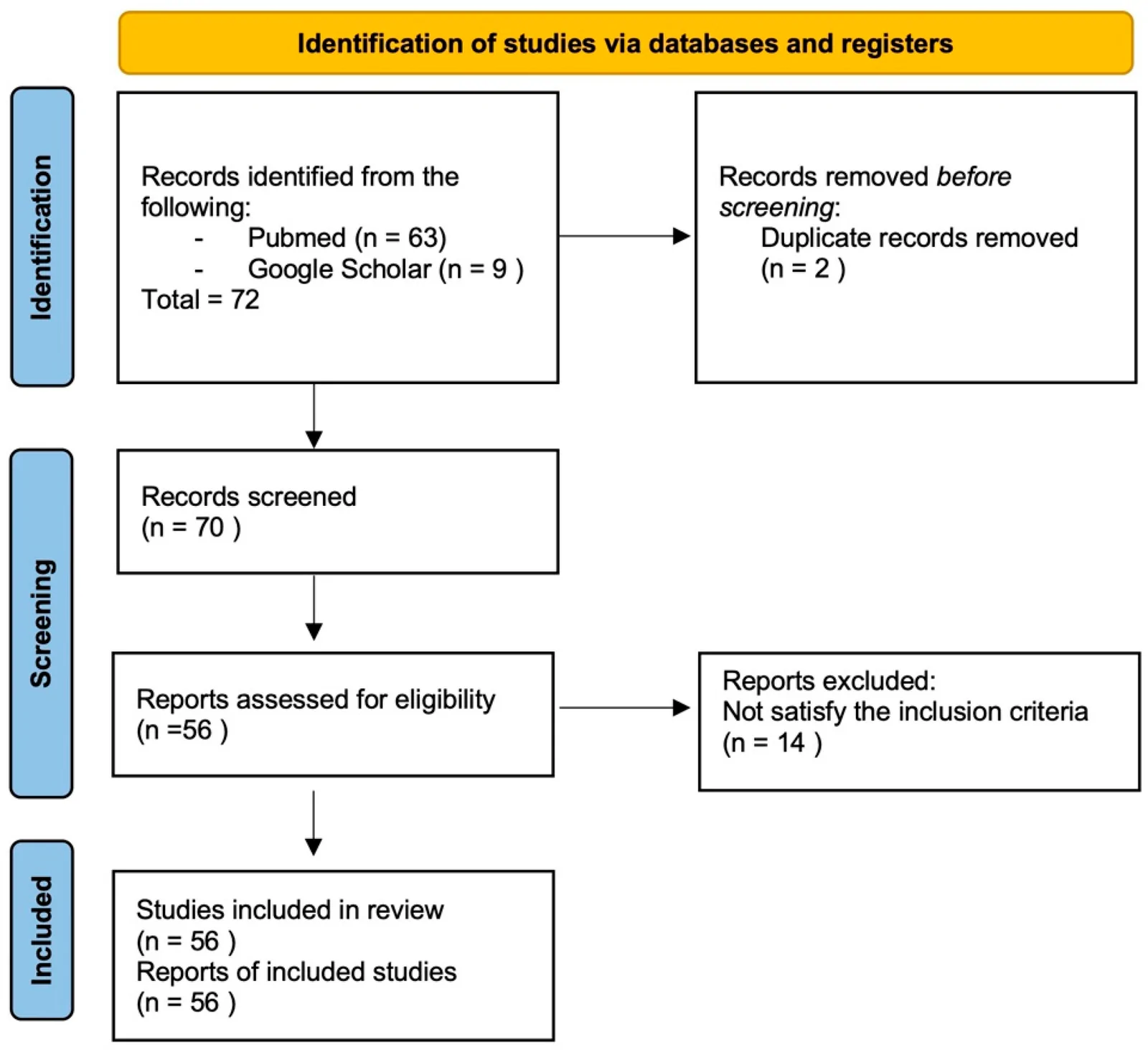
PRISMA diagram resembling the electronic database search and inclusion/exclusion review process.

We conducted an online systematic literature search according to the Preferred Reporting Items for Systematic Reviews and Meta-Analyses (PRISMA) recommendations (Figure 1; Table S1) [13]. We considered only articles available in the English language across the electronic databases from their respective dates of inception until 31 May 2025. The electronic databases PubMed and Google Scholar were examined. The search strategy utilized a combination of Medical Subject Headings (MeSH) terms and free-text-keywords including: “spinal artery aneurysm”, “spine”, “subarachnoid haemorrhage”, and “arterial dissection”. All the included articles were collected and analyzed independently by the authors and cross-checked for accuracy. To avoid missing relevant literature, the reference lists of eligible articles and relevant reviews were manually screened by the authors.

Studies were considered eligible according to the following inclusion criteria: (I) confirmed radiological diagnosis of isolated ruptured spinal aneurysm (anterior spinal artery, posterior spinal artery or artery of Adamkiewicz); (II) studies published in the English language. Exclusion criteria were as follows: (i) lack of relevant data; (ii) studies on unruptured spinal arterial aneurysms or on vertebral or intracranial artery aneurysms; (iii) studies on spinal arterial aneurysms associated with vascular malformation or other pathologies that increase hemodynamic stress (Moyamoya disease, vertebral artery occlusion, coarctation of the aorta, multiple aneurysms, spinal AVM, vasculitis, post-traumatic); (iv) studies not published in the English language; and (v) studies focusing on the pediatric population.

Literature screening was independently performed by two reviewers to eliminate irrelevant studies. Each relevant article was carefully evaluated to collect information; two reviewers independently evaluated the papers, and any disagreement was resolved through consensus or by consultation with a third author. The extracted data from the final included articles were collected into a standardized electronic database; the extracted variables included: first author, year of publication, age and sex, clinical presentation, aneurysm shape, spinal level, treatment, imaging outcome and clinical outcome.

Due to the rarity of isolated spinal aneurysms, the included literature consisted exclusively of retrospective case reports and small case series. To address the inherent risk of bias in these study designs, two reviewers qualitatively appraised the clinical detail and diagnostic certainty of each report. Studies lacking key diagnostic confirmation (e.g., clear angiography or intraoperative confirmation) or those with highly incomplete clinical data were excluded to ensure a minimal standard of data reliability.

Given the anticipated nature of the literature, a formal meta-analysis was not feasible due to substantial clinical heterogeneity. Consequently, a descriptive qualitative synthesis was performed. Categorical variables were summarized using frequencies and percentages.

## 3. Case Presentation

A 60-year-old Caucasian male was referred to our Department of Neurosurgery and initially presented with a 48 h history of lumbar and abdominal pain, which swiftly became associated with headache, photophobia, worsening neck pain and rigor nucalis. A complete clinical examination of sensory, motor and reflexes confirmed he had no neurological deficits. A non-contrast computed tomography scan (CT) of the head and spine demonstrated no evidence of SAH. The patient developed mild bladder dysfunction, so an urgent spine magnetic resonance imaging (MRI) was performed, suspecting a spinal vascular pathology. The MRI revealed a spinal subdural hematoma extending from the T9 to the T11 level, characterized by T1-isointense and T2-hyperintense signals. This collection was associated with mild compression of the spinal cord and slight, focal T2-hyperintensity of the cord signal, suggesting early suffering.

**Figure 2:**
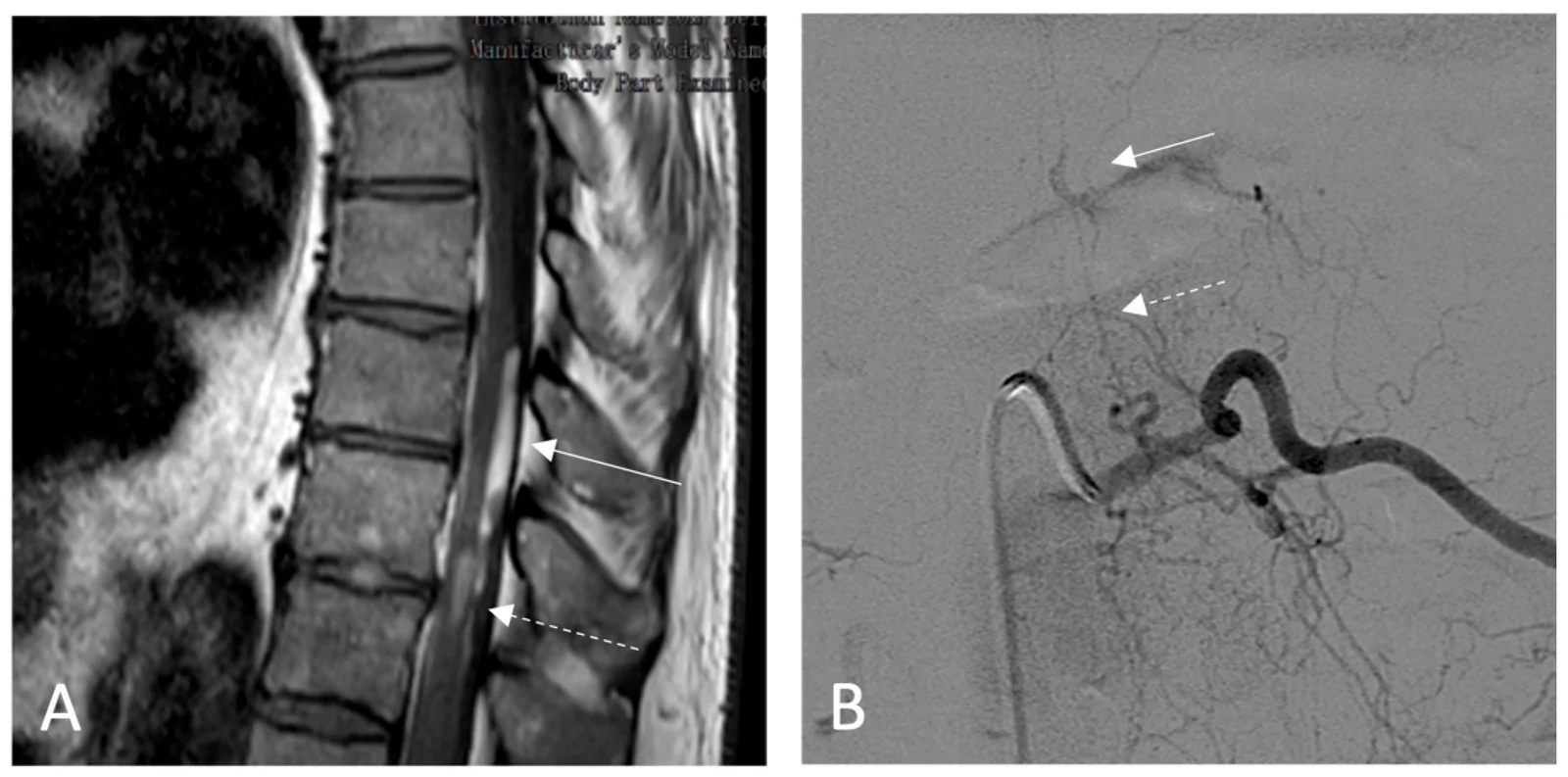
(**A**) Spinal MR, T1w sequence, sagittal plane demonstrates a subdural blood collection (solid arrow) in the posterior aspect of the canal, from T9 to T11 level, with an intradural component (dotted arrow) exerting slight cord compression. (**B**) Spinal Digital Subtraction Angiography on admission, AP view, left T11 intercostal artery injection, showing a small fusiform aneurysm (solid arrow) originating from a postero-lateral spinal branch (dotted arrow).

To outline the vascular anatomy, a digital subtraction angiogram (DSA) was performed, which demonstrated a fusiform dissecting aneurysm arising from the left T11 posterior spinal artery (Figure 2).

**Figure 3:**
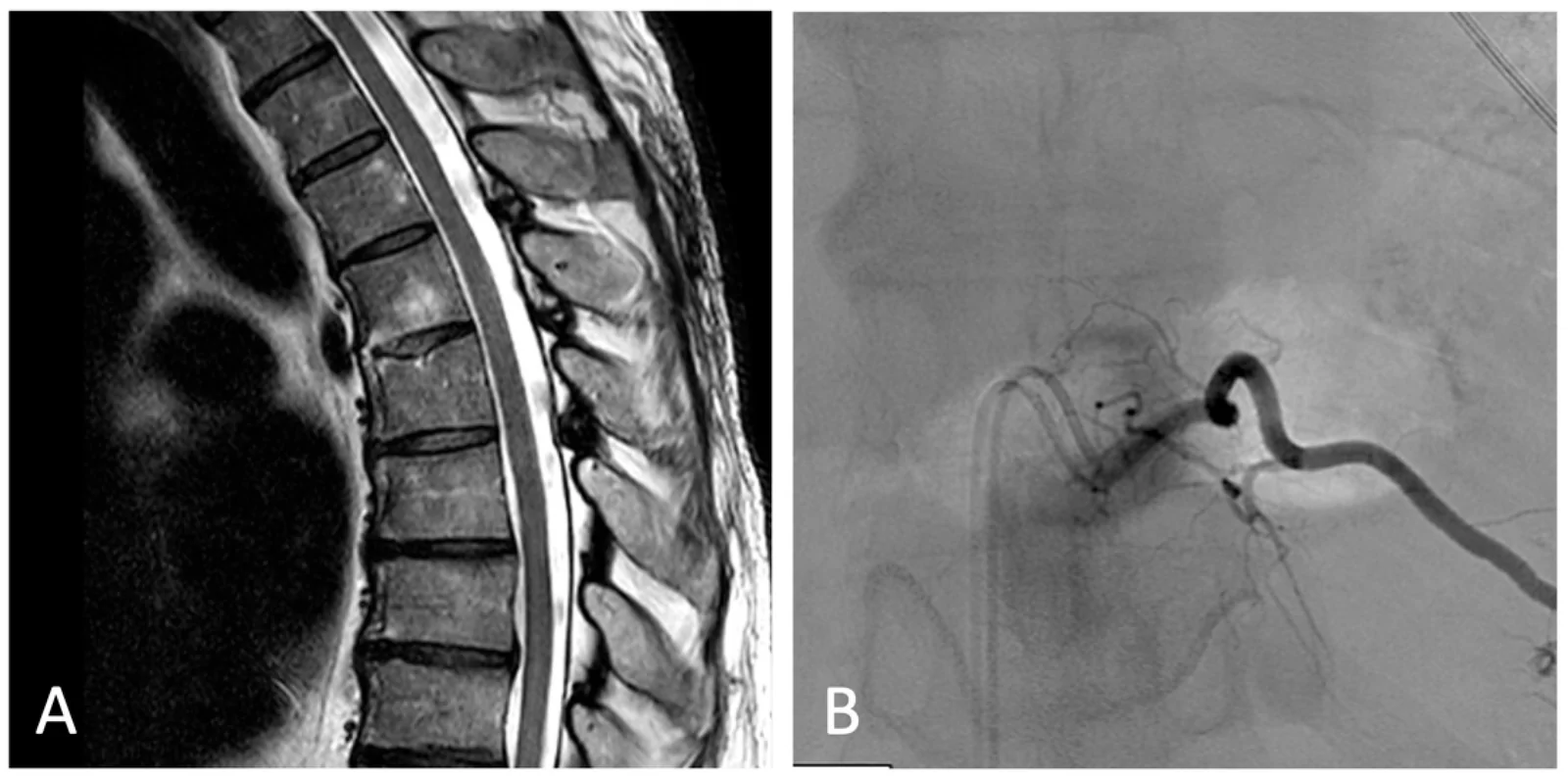
(**A**) Follow-up Spinal MR, T2w sequence, sagittal plane, shows complete reabsorption of the blood collections without residual myelopathy or post-ischemic cord lesions. (**B**) Spinal Digital Subtraction Angiography control 1 week later, AP view, left T11 intercostal artery injection shows spontaneous occlusion of the postero-lateral spinal branch and complete disappearance of the aneurysm.

The patient was managed conservatively; he had good clinical recovery with rehabilitation, the bladder dysfunction resolved within a few days and no new neurological complaints. The 1-week follow-up DSA showed disappearance of the aneurysm. Subsequent one-year-MRI investigations confirmed no residual aneurysm or other pathological features (Figure 3).

## 4. Results

A total of 72 articles were identified through the database searches, among which 56 articles met the inclusion criteria and were included in the analysis. Each relevant article was carefully evaluated to collect information on patient demographics, clinical features, treatment and outcomes. In our data, “surgical management” was defined as aneurysm microsurgical resection, clipping or wrapping and/or bypass; hematoma evacuation only was recorded as “conservative management.”

To our knowledge, there are slightly fewer than seventy cases of isolated rupture SAAs reported in the literature; excluding the present case, there are 29 reported cases of isolated PSAAs. We compare records of 69 cases of SAAs and summarize their demographic, radiological, clinical and outcome features (Table 1). The patients’ ages ranged from 16 to 88 years, and there was no significant gender predominance (48% male; 52% female). Primary symptoms varied depending on the level of the aneurysm with respect to the spinal cord; the presentation of high cervical tract ruptured SAA closely resembles that of ruptured intracranial aneurysms [2,4]. The most common presenting symptoms were headache and back pain (51%). Other common presenting symptoms were nausea, vomiting and/or neurologic deficits. Neurological deficits could include paraparesis, quadriplegia, and sphincter dysfunction due to cord compression. Usually, patients with thoracic or lumbar SAA present with abdominal or back pain (82%). Isolated aneurysms were located at the ASA (39%), at the PSA (42%) or at the Adamkiewicz artery (19%). The majority of SAAs were located at the thoracic level (68%), especially caudal to T10 or at the cervical level (28%).

The surgical treatment strategy was mentioned in the majority of the reports. In the data collected, patients with ASAA were managed conservatively (13/26; 50%), surgically (12/26; 46%) or by embolization (1/26; 4%). One patient died before any treatment. Patients with PSAA were managed conservatively (4/29; 14%), surgically (21/29; 72%) or underwent endovascular embolization (4/29; 14%). Patients with Artery of Adamkiewicz aneurysms (AAKA) were managed conservatively (8/12; 67%) or surgically (4/12; 33%). One patient died before any treatment.

Among the 69 patients for whom outcome data were available, 87% (60/69) had favorable outcomes. Four patients died (6%), and three patients (4%) had no motor recovery. Notably, in three of the four patients who died, SAA was located at the upper cervical spine level.

Regarding PSAAs, we found that one out of 21 patients died and 2/21 had no clinical improvement in the group treated surgically; 1/4 patient had no recovery in the group treated with endovascular management. The other 21/25 patients recovered successfully, regardless of the therapeutic approach chosen. All patients with PSAAs that worsened presented with severe symptoms.

## 5. Discussion

We report a case of conservative management for ruptured dissecting isolated PSAA. To date, fewer than one hundred cases of SAAs have been described in the literature (ruptured, unruptured and associated with other spinal vascular malformations); however, the true incidence and natural history of SAAs remain poorly defined because of limited reported cases [1,2,4]. The majority of SAAs are discovered because of their rupture. Due to their small caliber, the spinal vessels are infrequently affected by atherosclerosis, and aneurysms remain undiagnosed until they become symptomatic [3].

The rupture of an isolated SAA, as the etiology of spinal SAH, is much rarer; it is estimated that less than 1% of SAHs result from rupture of spinal aneurysms. Other studies reported an incidence of less than one spinal artery aneurysm in 3000 angiograms [4].

SAAs differ from intracranial aneurysms because they typically are pseudoaneurysms; they tend to be small in size and arise from the parent artery along its course rather than from arterial branching sites [2]. It is hypothesized that pathophysiological mechanisms contribute to the formation of both cerebral and spinal aneurysms; however, spinal arteries have a smaller caliber and they are less affected by atherosclerosis [4]. In fact, SAAs usually rupture secondary to a dissection, which explains their fusiform nature and the absence of a surgical neck [1,2,4]. SAAs most commonly affect the cervical proximal portion or the thoracolumbar junction. Geibprasert et al. also observed that PSAAs were predominantly isolated dissecting aneurysms [2,5]. It has been reported that risk factors such as hypertension and female sex seem to have a higher association with SAAs; however, other previous reviews reported an equal prevalence in middle-aged adults of both sexes, without any strong correlation between common SAH risk factors and SAAs considered solely [2,4,6].

Non-contrast spinal CT and spinal MRI are usually capable of revealing acute sSAH and suspecting a pathological vascular anatomy of the spinal cord [3]. However, because of the small caliber of these vessels, MRI and CT are usually not able to reliably detect these lesions and therefore their diagnosis requires a high degree of clinical suspicion. DSA is the gold standard in the examination of non-traumatic sSAH, especially when cerebral aneurysms are excluded [2,5]. Cervical aneurysms seem to be associated with worse outcomes compared to thoracolumbar aneurysms, likely because the blood often spreads into the cranium and intraventricular hemorrhage could occur [7]. However, unlike intracranial aneurysmal rupture, where vasospasm and acute hydrocephalus represent major life-threatening complications requiring aggressive monitoring, these phenomena are significantly less frequent in spinal SAH due to different CSF dynamics and lower localized blood volume. Nevertheless, when persistent meningeal irritation occurs, with appropriate patient selection, lumbar drainage can be a highly effective therapeutic tool to clear blood breakdown products and mitigate intracranial pressure [58].

Therapeutic approaches for ruptured SAAa involve surgery, endovascular treatment, or conservative management; each of these has its own set of advantages and risks. When planning the treatment, usually the etiology, location of the lesion, growth and distal flow are typically considered [8]. In the case of isolated PSAA, a posterior surgical approach is reasonably straightforward because the artery is located on the dorsolateral side of the spinal cord [7]. However, direct clipping of PSAAs is usually not feasible because most of these lesions occur along the intradural portion of arteries as they course toward the spinal cord, lack a surgical neck and are fusiform [4]. Moreover, it is known that preserving the parent artery during surgery is challenging and for this reason, intraoperative electrophysiologic monitoring is recommended. Endovascular treatment is usually difficult due to the small diameter of the artery that challenges access by microcatheter [7,9,10]. Endovascular procedures with selective coiling may be suggested when the aneurysm exhibits a saccular morphology and narrow neck. As Spetzler et al. suggested, “*If distal flow is absent at surgery or in angiographic images, the aneurysm may be resected and the parent vessel sacrificed or embolized by endovascular technique. If there is evidence of distal blood flow and direct clipping of the aneurysm is not possible, preservation of the parent vessel is essential. In these cases, the lesion can be reinforced with muslin wrapping, or it can be surgically resected by direct microvascular reconstruction*” [59].

Several authors have promoted surgery or endovascular embolization to treat PSAAs; however, radiological and intraoperative findings suggest that a wait-and-see strategy with angiographic follow-up may represent a valid alternative [1,10,11]. The majority of isolated PSAAs are fusiform and rupture secondary to a dissection. Several cases and clinical data also suggest PSAAs are prone to thrombosis because of the narrow, winding nature of these vessels and they often occlude spontaneously. Moreover, PSA have rich anastomoses with the pial network and the ASA, so that single-vessel occlusion is unlikely to disturb the circulation in the spinal cord. Although PSA has an abundant pial network, motor evoked potential deterioration is reported during temporary clipping of the PSA [9]. This reinforces the potential role of conservative management as a possible therapeutic strategy in selected patients [7]. Berlis et al. have proposed the mass effect of aneurysms or blood clots to be the only indication for surgical treatment. However, it is difficult to differentiate which aneurysm truly requires active treatment and which conservative management [9]. In most case reports, therapeutic management was delayed with respect to the clinical onset. At this stage, PSAA may already have been occluded by thrombosis. Repeating an early DSA follow-up may show the aneurysm decreased in size, suggesting that a closely monitored wait-and-see approach might be a reasonable therapeutic option for stable, asymptomatic cases. On the contrary, a growing PSAA is a red flag indicating rapid treatment is necessary.

The patient in our case was treated conservatively, adhering to a personalized medicine framework after a detailed personalized risk-benefit assessment. We carefully considered and integrated the aneurysm’s morphology on radiological imaging with the patient’s clinical presentation at onset and, crucially, his individual life and occupation needs.

First, the patient presented with a classical fusiform aneurysm, lacking a surgical neck and mild neurological deficits at the onset. Given the extremely small caliber of the PSA, the presence of distal flow and the absence of cord compression, the risks of PSAA rupture associated with surgical treatment would exceed the predictable benefits of such a surgical procedure [10]. Moreover, the patient works as a martial arts instructor and the preservation of sensory function (the conscious appreciation of fine touch and discrimination, conscious proprioception and vibratory sensations) was a priority for him. Because this rare pathology lacks standardized guidelines, clinical decisions must be highly individualized. Consequently, we discuss the patient’s personal treatment goals and lifestyle expectations. A closely monitored tailored wait-and-see strategy was preferred and ultimately it proved successful. An early follow-up DSA demonstrated complete spontaneous regression of the lesion. We chose conservative management due to the mild neurological deficits and in order to preserve the dorsal column medial lemniscus pathway; the goal was to avoid risking a surgical deficit along this pathway, which would have represented an unacceptable, non-beneficial outcome for this specific patient’s career and daily activities.

Finally, the publication of case reports may be affected by positive outcome bias, regardless of whether surgical treatment was performed. For this reason, it is hazardous to draw rigid, universal conclusions about the best treatment based solely on sporadic case reports. Instead, we advocate for a personalized clinical approach, emphasizing effective communication of individualized risk-benefit profiles to select the most appropriate, tailored management strategy for each unique patient [11].

## 6. Conclusions

It is difficult to distinguish which SAAs truly require active treatment; ruptured SAAs are prone to spontaneous thrombosis and often radiological and intra-operative findings suggest that angiographic follow-up may represent a valid guide in their management. In the absence of neurological defects, with the presence of distal flow and without evidence of aneurysm growth, a conservative approach may be cautiously considered as an alternative strategy. However, given the unpredictable natural history of these lesions, close clinical and radiological monitoring remains mandatory, and active intervention should be promptly executed if any clinical or anatomical deterioration occurs. Understanding this rare pathology is critical to clarify its natural history and to guide diagnosis. The therapeutic strategy should be tailored through a comprehensive, personalized risk assessment that integrates high-resolution radiological imaging, clinical presentation and the patient’s individual lifestyle and personal history.

## Figures and Tables

**Table 1:** Summary of case reports describing patients with ruptured isolated SAAs.

	Author, Years	Age, Sex	Clinical Presentation	Shape	Aneurysm Level	Treatment	Imaging Outcome	Clinical Outcome
	Anterior Spinal Artery Aneurysm							
1	Turrini et al., 2021 [2]	64/M	Dizziness and numbness, headache and vomiting.	NA	C1	Hematoma evacuation, surgical resection	Aneurysm regression	General weakness, gradually recovered
2	Abdalkader et al. 2021 [14]	70/M	Sudden-onset headache and vomiting	NA	C2	Wait-and-see	NA	Good clinical recovery
3	Cobb et al.; 2020 [3]	36/F	Acute low back pain and paraplegia	Fusiform dissecting	T11	Embolization (onyx)	NA	No recovery from paraplegia
4	Roka et al., 2019 [15]	30/F	Suboccipital headache, vomiting, vertigo, gait abnormality, and nuchal rigidity	NA	C0	Wait-and-see	NA (refused)	Good clinical recovery
5	Dabus et al., 2018 [16]	65/M	NA	NA	C0	Wait-and-see	Aneurysm regression	Good clinical improvement
6	Ren et al., 2018 [17]	57/F	Acute severe headache	Fusiform	C1	Hematoma evacuation, surgical resection	Aneurysm regression	Good clinical recovery
7	Ren et al., 2018 [17]	27/F	Progressive bilateral pain of her lower limbs	NA	L1	Hematoma evacuation, surgical resection	Aneurysm regression	Good clinical recovery
8	Dabus et al., 2017 [16]	60/M	Severe headache and neck and back pain	NA	T12	Wait-and-see	Spontaneous resolution	Good clinical recovery
9	Nakhla et al., 2016 [18]	88/F	Severe global headache and neck pain	Saccular	C5–C6	Wait-and-see	NA	Good clinical recovery
10	Boeris et al., 2016 [19]	22/M	Severe sudden-onset head and neck pain after a fight	Fusiform	C4	Wait-and-see	Spontaneous resolution	Good clinical recovery
11	Sung et al., 2015 [4]	74/M	Gradual progressive headache and confusion requiring intubation	Saccular	T1	Hematoma evacuation, surgical resection	Aneurysm regression	Good clinical recovery
12	Romero et al., 2014 [20]	72/F	Cervical and back pain, headache, neck stiffness	Fusiform, dissecting	T10	Wait-and-see	MRI studies showed a decrease in the size	Good clinical recovery
13	Romero et al., 2014 [20]	37/F	Sudden onset of thoracic pain, cervical pain; headache; neck stiffness	NA	T3	Wait-and-see	Spontaneous resolution	Good clinical recovery
14	Pahl et al., 2014 [21]	43/F	Headache, vomiting, GCS 8	Saccular	C2	Wait-and-see	Aneurysm regression	Good clinical recovery
15	Seerangan and Narayanan, 2012 [22]	47/M	Back pain, left lower extremity weakness with bowel and bladder disturbances	NA	T8–T9	Hematoma evacuation; wait-and-see	NA	Minimal motor recovery of left leg
16	Takashima et al., 2012 [23]	84/M	Quadriplegia	NA	C1	NA	NA	Death from respiratory dysfunction
17	Karakama et al., 2010 [24]	51/M	Neck pain, headache, loss of consciousness	NA	C1	Wait-and-see	Aneurysm regression	Good clinical recovery
18	Peltier et al., 2010 [25]	38/F	Back pain, leg weakness, meningism	Fusiform	T12–L1	Wait-and-see	Aneurysm regression	Leg motor recovery, sphincter tone regained
19	Pollok et al., 2009 [26]	55/F	Headache, loss of consciousness	Fusiform	C1–C2	Wrapping	NA	Good clinical recovery
20	Longatti et al., 2008 [10]	54/F	Abdominal pain, vomiting, nuchal rigidity, leg weakness	NA	T9; T12	Wait-and-see	Aneurysm regression	Good clinical recovery
21	Massand et al., 2005 [27]	73/M	Back pain, bilateral leg pain, headache	Dissecting aneurysm	T6–T7	Hematoma evacuation, surgical resection	Aneurysm regression	Good clinical recovery
22	Massand et al., 2005 [27]	54/M	Sudden-onset back pain, radicular pain, bilateral lower extremity weakness	Dissecting aneurysm	T12	Hematoma evacuation, surgical resection	Aneurysm regression	Good clinical recovery
23	Kawamura et al., 1999 [28]	42/M	Headache	NA	NA	Surgical clipping	NA	Good clinical recovery
24	Smith et al., 1986 [29]	29/M	NA	Saccular	T12–L1	Surgical clipping	NA	Mild tibialis anterior paresis
25	Moore et al., 1982 [30]	30/F	Headache, right hemiplegia	Saccular	C1	Surgical clipping	Aneurysm regression, ASA preserved	Mild right hemiparesis
26	Vincent, 1981 [31]	30/F	NA	NA	C1–C2	Surgical clipping	NA	Mild spastic hemiparesis
27	Leech et al., 1976 [32]	25/F	NA	Saccular	T7	Surgical clipping	Aneurysm regression	Mild neurological deficits
	Artery of Adamkiewicz Aneurysms							
1	Limaye et al., 2021 [33]	43/F	Low back pain and paraesthesia	Dissecting aneurysm	T12	Wait-and-see	Spontaneous occlusion	Good clinical recovery
2	Aljuboori et al. 2018 [34]	78/M	Back pain and lower limb weakness	Fusiform	T9	Surgical clipping	NA	Good clinical recovery
3	Agarwal et al., 2017 [35]	47/F	Low back pain	NA	T10	Wait-and-see	Spontaneous occlusion	Good clinical recovery
4	Aguilar-Salinas et al., 2017 [36]	54/F	Lower extremity weakness and headache	NA	T8	Wait-and-see	Spontaneous occlusion	Good clinical recovery
5	Doberstein et al, 2016 [37]	59/M	Back pain, lower extremity weakness	NA	T11	Wait-and-see	Spontaneous occlusion	Good clinical recovery
6	Son et al., 2013 [38]	45/F	Headache, low back pain, nausea	NA	T12	Wait-and-see	Spontaneous resolution; MRI intradural fluid collection	Good clinical recovery
7	Lihosci et al., 2011 [39]	60/F	Headache, low back and left lower extremity pain	NA	T12	Wait-and-see	Spontaneous resolution	Good clinical recovery
8	Gonzales et al., 2005 [40]	30/M	Back pain, paresthesias, lower extremities	NA	T11	Hematoma evacuation, surgical resection	Aneurysm regression	Good clinical recovery
9	Massand et al., 2005 [27]	30/M	Back pain, paraparesis, and paresthesias	NA	T11	Hematoma evacuation, surgical resection	Aneurysm regression	Good clinical recovery
10	Berlis et al., 2005 [9]	69/F	Abdominal pain, paraplegia, anesthesia, nuchal rigidity, headache	NA	T12–L1	Wait-and-see	Spontaneous resolution	Good clinical recovery
11	Berlis et al., 2005 [9]	48/M	Back pain, vomiting, walking impairment	NA	T12–L1	Wait-and-see	Spontaneous resolution	Residual paresthesias in right foot, episodic paresis
12	Vishteh et al., 1997 [41]	30/M	Low back pain, headache	NA	T11	Hematoma evacuation, surgical resection	Aneurysm regression	Good clinical recovery
13	Garcia et al., 1979 [42]	34/F	Paraplegia, headache	NA	T6	None, discovered post-mortem	Death	Death
	Posterior Spinal Artery Aneurysm							
1	Kee et al., 2025 [43]	60/F	Abdominal pain, lower limb weakness, urinary retention	Fusiform	T2	Hematoma evacuation, surgical clipping	Not performed	Paraplegic
2	Kee et al., 2025 [43]	66/F	Lower extremity weakness, back pain	NA	T12	Endovascular coil embolization	Aneurysm regression	Mild residual left lower extremity paresis and paresthesias
3	Cadieux et al., 2021 [6]	54/F	Headache and interscapular back pain, cute, complete flaccid paraplegia	Fusiform	T2	Hematoma evacuation, surgical resection	Aneurysm regression	General weakness, gradually recovered
4	Tenorio et al., 2021 [44]	49/NA	Abdominal pain, back pain radiating to the thighs, bilateral lower extremity paralysis.	Saccular	T11	Hematoma evacuation, surgical resection	Aneurysm regression	General weakness, gradually recovered
5	Takebayashi et al., 2019 [7]	67/F	Nausea, low back pain and right pain with paresis	Fusiform	T10	Hematoma evacuation, Surgical resection	Aneurysm regression	Good clinical recovery
6	Dabus et al., 2017 [16]	62/M	Back pain and paresthesia lower extremity	NA	T6	Wait-and-see	Unchanged, no bleeding	Good clinical recovery
7	Takata et at, 2016 [45]	72/M	Back pain	NA	T9	Hematoma evacuation, surgical clipping	Aneurysm regression	Good clinical recovery
8	Horio et al., 2016 [11]	84/M	Left hemiplegia	Fusiform	T12	Hematoma evacuation, surgical resection	Aneurysm regression	Mild neurological deficits
9	Ronchetti et al., 2016 [46]	54/M	Severe back pain and vomiting	Fusiform	T10	Surgical resection	Aneurysm regression	Good clinical recovery
10	Ronchetti et al., 2015 [46]	51/F	Neck and back pain, headache, bilateral leg numbness, difficulty walking	Pseudoaneurysm	T4	Surgical resection	Aneurysm regression	Good clinical recovery
11	Ronchetti et al., 2015 [46]	68/M	Mid-back pain radiating to neck, headache	NA	T1	Embolization	Aneurysm obliteration	Good improvement of symptoms
12	Bell et al., 2014 [12]	68/F	Severe back pain	Psudoaneurysm	T5	Hematoma evacuation, surgical resection	Aneurysm regression	Good clinical recovery
13	Johnson et al., 2014 [47]	16/NA	Headache, neck pain and muscle spasms.	Fusiform	C5–C6	Hematoma evacuation, surgical resection	Aneurysm regression	Good clinical recovery
14	Van Es et al. 2013 [48]	68/M	Intrascapular back pain radiating to lumbar region, headache, nausea	NA	T4	Wait-and-see (patient refusal)	Unchanged, no bleeding	Good clinical recovery
15	Van Es et al. 2013 [48]	62/F	Headache, back pain, walking difficulty	NA	T12–L1	Hematoma evacuation, surgical resection	Aneurysm regression	Good clinical recovery
16	Kim and Choi, 2012 [8]	52/M	Back and abdominal pain, mild leg weakness, spinal SAH	Dissecting	T7	Surgical clipping	Aneurysm occlusion	Good clinical recovery
17	Tanweer et al., 2012 [49]	67/F	Paraplegia	Pseudoaneurysm	T10	Embolization	Aneurysm obliteration	Improved sensory; no motor recovery
18	Shankar et at, 2012 [50]	72/F	Back pain	Fusiform	L2	Embolization	Aneurysm obliteration	Good clinical recovery
19	Sato et al., 2012 [51]	67/F	Back pain, paraparesis, spinal SAH	NA	T8	Wait-and-see	NA	Good clinical recovery
20	Geibprasert et al. 2010 [5]	43/M	Headache and interscapular back pain;	Fusiform; dissecting	T4	Hematoma evacuation, surgical resection	NA	Good clinical recovery
21	Cavusoglu et al. 2010 [52]	27/F	Headache and neck pain	NA	C1	Surgical clipping	Aneurysm occlusion	Good clinical recovery
22	Kocak et al. 2006 [53]	54/F	Severe headache, intracranial SAH	Saccular	C2	Hematoma evacuation, surgical resection	NA	Rebled and death
23	Nemecek et al. 2006 [54]	55/M	Paraplegia, headache	Fusiform	T12	Hematoma evacuation, surgical resection	NA	Improved sensory; no motor recovery
24	Massand et al. 2005 [27]	69/M	Back and leg pain	Fusiform	L1	Hematoma evacuation, surgical resection	NA	Good clinical recovery
25	Massand et al. 2005 [27]	54/M	Back and leg pain	Fusiform	T12	Hematoma evacuation, surgical resection	NA	Good clinical recovery
26	Berlis et al. 2005 [9]	62/F	Headache, vomiting, neck and back pain	NA	T5	Hematoma evacuation, Surgical clipping	Aneurysm occlusion	Good clinical recovery
27	Caglar et al., 2005 [55]	74/F	Lower back pain radiated to the right leg	Partially thrombosed	T12	Hematoma evacuation, surgical resection	NA	Good clinical recovery
28	Goto et al., 1988 [56]	52/M	Headache, stupor	NA	C2	Hematoma evacuation, surgical resection	NA	Good clinical recovery
29	Henson & Croft, 1956 [57]	51/M	Headache and neck pain	NA	C1	Wait-and-see	NA	Died
	Present case	60/M	Lumbar and abdominal pain, headache	Fusiform	T11	Wait-and-see	Aneurysm occlusion	Good clinical recovery

## Data Availability

All data generated or analyzed during this study are included in the published paper. The review protocol was not registered.

## Supplementary Materials

The following supporting information can be downloaded at https://www.mdpi.com/article/10.3390/jpm16090462/s1, Table S1: PRISMA_2020_checklist.

## Abbreviations

CT	Computed tomography
DSA	Digital subtraction angiography
ASAA	Anterior spinal artery aneurysm
PSAA	Posterior spinal artery aneurysm
AAKA	Artery of Adamkiewicz aneurysm
SAA	Spinal artery aneurysm
sSAH	Spinal subarachnoid hemorrhage
